# Carbon dioxide-enriched atmosphere diminished the phytotoxicity of neodymium in wheat (*Triticum aestivum* L.)

**DOI:** 10.3389/fpls.2025.1521460

**Published:** 2025-04-28

**Authors:** Ahmed M. Saleh, Maha S. A. Haridy, Afrah E. Mohammed, Lewis Ziska, Modhi O. Alotaibi, Ahmed M. A. Khalil, Mahmoud M. Y. Madany, Hamada Abd Elgawad, Hanaa E. A. Amer

**Affiliations:** ^1^ Biology Department, Faculty of Science at Yanbu, Taibah University, Yanbu El-Bahr, Saudi Arabia; ^2^ Botany and Microbiology Department, Faculty of Science, University of Cairo, Giza, Egypt; ^3^ Central Lab of Organic Agriculture, Agricultural Research Center (ARC), Giza, Egypt; ^4^ Department of Biology, College of Science, Princess Nourah bint Abdulrahman University, Riyadh, Saudi Arabia; ^5^ Microbiology and Immunology Unit, Natural and Health Sciences Research Center, Princess Nourah Bint Abdulrahman University, Riyadh, Saudi Arabia; ^6^ Environmental Health Sciences, Mailman School of Public Health, Columbia University, New York, NY, United States; ^7^ Environmental and Biomaterial Unit, Natural and Health Sciences Research Center, Princess Nourah Bint Abdulrahman University, Riyadh, Saudi Arabia; ^8^ Biology Department, Faculty of Science, Taibah University, Al-Madinah Al-Munawarah, Saudi Arabia; ^9^ Department of Botany and Microbiology, Faculty of Science, Beni-Suef University, Beni-Suef, Egypt

**Keywords:** neodymium, elevated CO_2_, wheat, photosynthesis, antioxidants

## Abstract

**Introduction:**

Neodymium (Nd), a rare earth element (REEs), is widely utilized in industry. Although the detailed biological role of Nd in plant biology is unclear, recent reports have noted its oxidative phytotoxicity at concentrations higher than 200 mg kg^-1^ soil. At present it is unclear if these detrimental effects could be offset by the global rise in atmospheric carbon dioxide concentration ([CO_2_]) which has been shown to enhance photosynthesis and growth in a wide range of C3 plant species.

**Methods:**

To assess any amelioration effects of [CO_2_], a phytotoxic dose of Nd (III) was given to wheat grown under two scenarios of atmospheric CO_2_, ambient levels of CO_2_ (aCO_2_, 420 ppm) and eCO_2_ (620 ppm) to assess growth and photosynthesis.

**Results and discussion:**

Our results suggest that at ambient [CO_2_], Nd treatment retarded wheat growth, photosynthesis and induced severe oxidative stress. In contrast, eCO_2_ reduced the accumulation of Nd in wheat tissues and mitigated its negative impact on biomass production and photosynthesis related parameters, i.e., photosynthetic rate, chlorophyll content, Rubisco activity and photochemical efficiency of PSII (Fv/Fm). Elevated [CO_2_] also supported the antioxidant defense system in Nd-treated wheat, enhanced production of enzymatic antioxidants, and more efficient ascorbate-glutathione recycling was noted. While additional data are needed, these initial results suggest that rising [CO2] could reduce Nd-induced oxidative stress in wheat.

## 1 Introduction

Extensive human activities are slowly increasing atmospheric carbon dioxide concentration [CO_2_] in the atmosphere (Nunes, 2023). In addition to its role as a greenhouse gas, [CO_2_] is the primary source of carbon for plant photosynthesis and its increase has been associated with improved growth and fecundity for a number of C3 plant species, including crops (AbdElgawad et al., 2022b, 2023; Ziska, 2022).

Although plant response to [CO_2_] per se is acknowledged as beneficial, interactions with other abiotic parameters, including heavy metals, is still unclear. Work on arsenic in rice has suggested that elevated [CO_2_] may increase arsenic concentration in the rice grain (Wang et al., 2023); conversely, elevated [CO_2_] attenuated the toxicity of NiO-nanoparticles on wheat (Saleh et al., 2019); arsenic oxide nanoparticles on *Zea mays* (Selim et al., 2021); and silicon dioxide nanoparticles on pea (Shabbaj et al., 2021).

Rare earth elements (REEs), which include scandium, yttrium, and the 15 lanthanides, possess distinctive properties that make them highly valuable across various industries and products. REEs are integral to high-tech devices such as rechargeable batteries, computers, mobile phones, medical imaging equipment, LCD screens, and radar systems (Hu et al., 2016). Additionally, in agriculture, these elements are used in small concentrations as fertilizers; however, elevated levels can negatively impact edible horticultural crops, leading to environmental and human health risks. The bioaccumulation of REEs has been shown to adversely affect aquatic organisms (Revel et al., 2025), as well as terrestrial life, including plants, animals, and humans (Wang et al., 2024; Petrini et al., 2025). Therefore, it is important to investigate the potential hazards associated with REEs accumulation under different environmental conditions. Understanding these dynamics is critical for promoting safe and responsible use of these elements in various applications.

The rising levels of atmospheric [CO_2_] may enhance plant growth and resilience, potentially counteracting the adverse effects of rare earth elements (REEs) however, its role to compensate, or exacerbate, any REE in plant effects has not been evaluated. Neodymium (Nd) is a lanthanide and falls within the category of rare earth elements (REEs). Available reports indicate that Nd concentrations in uncontaminated soils can range from 5.8 to 53 mg kg^-1^ (Carpenter et al., 2015; Patra et al., 2020; Wang et al., 2020). Although its biological role of Nd is still obscure, phytotoxicity of Nd has been reported (Zhao et al., 2019). The phytotoxicity of a high Nd dose (319.00 mg kg^-1^) on plant growth was stronger compared to other REEs, i.e., Sm, Tb, Dy and Er (Carpenter et al., 2015). Therefore, more investigations are needed to address Nd effect on some important crops in the human nutrition such as wheat (*Triticum aestivum*) which has been chosen for the current study.

Wheat is a crucial staple in the human diet, particularly in developing countries (Rizvi et al., 2020). While there is established evidence regarding the negative effects of heavy metals such as bismuth, cadmium, copper, and zinc on various biological processes in wheat (Mohammed et al., 2023, Athar and Ahmad, 2002; Hammami et al., 2022), the interaction between rising atmospheric CO_2_ levels and heavy metal contamination remains inconsistent. For instance, a Free-Air CO_2_ Enrichment (FACE) study found that elevated CO_2_ levels resulted in reduced copper (Cu) concentrations but increased cadmium (Cd) concentrations in both the shoots and grains of wheat and rice grown in contaminated soils (Guo et al., 2011).

While detailed investigations on neodymium (Nd) exposure in relation to elevated [CO_2_] per se are currently limited, this study aims to fill that gap. Therefore, the objectives currently were to assess the redox status of wheat under Nd stress, both with and without supplemental CO_2_, and to examine the impact on key physiological processes, including photosynthesis. To evaluate the accumulation of Nd in plant tissues, along with key growth parameters and photosynthetic efficiency. To monitor changes in wheat stress markers and analyze the activity of molecular and enzymatic reactive oxygen species (ROS) scavengers, as well as the components of the ascorbate-glutathione cycle. Through this comprehensive assessment, authors aimed to deepen our understanding of how elevated CO_2_ can influence wheat’s response to Nd exposure, providing valuable insights into both plant physiology and environmental interactions.

## 2 Methods

### 2.1 Plant growth and treatments

Wheat (*Triticum aestivum* L., cv Giza 112) grains were surface sterilized using sodium hypochlorite (5% v/v) for 20 minutes before being seeded in pots with an artificial soil that were 15 cm deep and 13 cm in diameter. For 1 g of air-dried soil, the following was the soil composition: carbon (11.7 mg), ammonium N (1.1 mg), nitrate nitrogen (14.8 mg), phosphorus (9.4 mg), and humidity (0.33 g water). Four different set-ups were used to arrange the plants: 1) ambient CO_2_ (aCO_2_, 420 ppm CO_2_; 2) 200 mg Nd^3+^kg^-1^ soil under aCO_2_, (Nd); 3) elevated CO_2_ (eCO_2_, 620 ppm) and 4) 200 mg Nd^3+^kg^-1^ soil under eCO_2_, (Nd+eCO_2_). A preliminary experiment determined the Nd (III) concentration, and the eCO_2_ level was determined using the IPCC-SRES B2-state estimate of eCO2 for the year 2100 (Murray and Ebi, 2012). Nd was applied in the form of NdCl_3_.6H_2_O. Pots were kept in a growth chamber at a temperature of 21/18°C with a photoperiod of 16/8h (photosynthetically active radiation of 350 µmol photons m^-2^ s^-1^) and 60% humidity. In the growth room, the pots were placed at random and regularly watered. The experiment was repeated, but this time the two CO_2_ levels in the cabinets were switched. The plants were collected four weeks after the seeds were sown, the shoots and roots’ fresh and dried weights were measured. The plant material was transported in liquid N and stored at -80°C. In order to conduct further research, soil samples were collected and stored at -20°C in an ice box. Five replicates of each treatment (pot) were performed.

### 2.2 Photosynthesis related parameters

According to (Abdelgawad et al., 2015), light-saturated photosynthetic rate (Asat) and stomatal conductance (gs) were measured from the youngest-fully enlarged leaf using the LI-COR LI-6400, a device made by LI-COR Inc. in Lincoln, Nebraska, USA. Photosynthesis was evaluated at light intensity of 1,500 μmol m^−2^ s^−1^. The CO_2_ concentration in the leaf chamber was maintained at 420 µmol mol^–1^ for the aCO_2_ group and 620 µmol mol^–1^ for the eCO_2_ group, with the temperature controlled at 25 ± 0.5°C. photosynthetic pigment concentration was extracted in acetone and quantified according to Porra et al. (1989). Using a fluorimeter (PAM2000, Walz, Effeltrich, Germany) over a 30-minute period, chlorophyll fluorescence was calculated from the dark-adapted leaves.

### 2.3 Determination of neodymium accumulation

The dried plant samples were treated in 13 M nitric acid at 185°C for 25 minutes for Nd extraction (Agusa et al., 2005). Inductively coupled plasma mass spectrometry (ICP-MS; model 820-MS) with a glass nebulizer operating at 0.4 mL/minute was being used to determine the element concentration. For calibration curves, external standards with concentrations ranging from 1 to 600 g/L were created. Yttrium was also introduced to regulate nebulizer efficacy as an internal standard during extraction. In 0.23 M nitric acid, standard minerals were created.

### 2.4 Oxidative stress markers

Malondialdehyde (MDA) was identified in plant samples using the thiobarbituric acid method as a marker for lipid peroxidation (Hodges et al., 1999). Xylenol orange method was used for determining H_2_O_2_ in plant samples after extraction in 0.1% trichloroacetic acid (Jiang et al., 1990).

### 2.5 Total antioxidant capacity and antioxidant metabolites

Plant materials that were extracted in 2 mL of 80% ice-cold ethanol by a MagNALyser were ground using liquid-N_2_. The ferric reducing/antioxidant power (FRAP) assay was used to calculate the total antioxidant capacity (TAC) of the extracts and was performed at 600 nm on a microplate reader, using Trolox as a standard (Benzie and Strain, 1999). HPLC analysis was used to evaluate the levels of reduced ascorbate (ASC) and reduced glutathione (GSH) (Potters et al., 2004). The total ascorbate (ASC+DHA) and glutathione (GSH+GSSG) concentration was assessed after reduction by DTT. Phenolic compounds were extracted from plant samples with 80% ethanol. After that, measurements of polyphenols (Zhang et al., 2006) and flavonoids (Chang et al., 2002) were performed using a spectrophotometer (Shimadzu UV-Vis 1601 PC, Japan). After hexane extraction, extracts were collected (CentriVap concentrator, Labconco, Kansas, USA; normal phase conditions, Particil Pac 5 m column material, length 250 mm, i.d. 4.6 mm) for tocopherols quantification by HPLC (Shimadzu, Hertogenbosch, the Netherlands; normal phase conditions). Dimethyl tocol was used as an internal standard at 5 ppm.

### 2.6 Antioxidant enzymes and glutathione-S-transferase

For the extraction of antioxidant enzymes, MagNALyser (Roche, Vilvoorde, Belgium) was used along with Triton X-100 (0.25%, v/v), polyvinyl pyrrolidone (10%, w/v), ASC (1 mM) and phenylmethylsulfonyl fluoride (1 mM) in a potassium phosphate buffer (50 mM, pH 7.0). After centrifugation (10 minutes, 13000 rpm, 4°C) the supernatant was obtained and the activities of catalase (CAT, EC 1.11.1.6), peroxidase (POX, EC 1.11.1), superoxide dismutase (SOD, EC 1.15.1.1), ascorbate peroxidase (APX, EC 1.11.1.11), glutathione peroxidase (GPX, EC 1.11.1.9), glutathione reductase (GR, EC 1.6.4.2), dehydroascorbate reductase (DHAR, EC 1.8.5.1) and monodehydroascorbate reductase (MDHAR, EC 1.6.5.4) were measured. The inhibition of nitroblue tetrazolium reduction at 560 nm was used to measure the activity of SOD (Dhindsa et al., 1982). The method recommended by Kumar and Khan (1982) was employed to evaluate the pyrogallol oxidation in order to determine POX activity. According to Aebi (1984), the dissociation of H_2_O_2_ at 240 nm was used to test the activity of CAT. The measurements of APX, DHAR, MDHAR, and GR activity followed the guidelines provided by Murshed et al. (2008). The NADPH oxidation decrement at 340 nm as published by (Drotar et al., 1985) was used to estimate the activity of GPX. Glutathione-S-transferase (GST, EC 2.5.1.18) was evaluated using potassium phosphate buffer (50 mM, pH 7.0) (Diopan et al., 2008). The method described by Lowry et al. (1951) was employed to determine the amounts of soluble proteins in the extracts.

### 2.7 Statistical analyses

Experiments were carried out using a completely randomized block design. The Statistical Analysis System (SPSS Inc., Chicago, IL, USA) was used to analyze the data. The Kolmogorov-Smirnov and Levene’s tests were used to determine data normality and variance homogeneity. The data were all subjected to Two-way analysis of variance (ANOVA). Tukey’s Test (p = 0.05) was used as a *post hoc* test for mean separation. Each experiment was replicated five times (n = 5).

## 3 Results

### 3.1 Growth and photosynthesis

Neodymium treatment significantly decreased the FW and DW of wheat plants by 57 and 60%, respectively, relative to the control (**Table 1**). In contrast, eCO_2_ alone showed a fertilization effect on wheat plants, significantly improving biomass production, by 43 and 40% for FW and DW, respectively (**Table 1**). The combined treatment (Nd+eCO_2_) showed a moderate increase in FW and DW relative to Nd alone, but significantly less than that of the elevated [CO_2_] treatment (**Table 1**). In parallel with FW and DW, the rate of photosynthesis in wheat plants treated with Nd alone declined sharply (- 44%) relative to the control (**Table 1**). Such reduction was concomitant with a significant decrease in chlorophyll content, chlorophyll fluorescence, stomatal conductance and Rubisco activity. On the other hand, eCO_2_ alone resulted in a significant improvement in photosynthetic rate, chlorophyll content and chlorophyll fluorescence at 24, 29 and 7.5% respectively, but had no significant effect on stomatal conductance or Rubisco activity compared to control (**Table 1**). The co-application of eCO_2_ on Nd-stressed plants significantly enhanced the rate of photosynthesis, chlorophyll content, Rubisco activity and photochemical efficiency of PSII (Fv/Fm) relative to Nd alone. A two Way ANOVA indicated significant interaction of Nd and eCO_2_ in the FW, DW, Photosynthesis, GS and the pigments except CHLa (**Supplementary Data Sheet S1**).

**Table 1:** Effect of neodymium (Nd), elevated CO_2_ (eCO_2_) and their synchronous application (Nd + eCO_2_) on the fresh (FW) and dry (DW) biomasses and the photosynthesis related parameters in wheat plants.

	Control	Nd	eCO_2_	Nd+eCO_2_
**FW** (g plant^-1^)	1.05 ± 0.06 c	0.45 ± 0.06 a	1.5 ± 0.09 d	0.63 ± 0.09 b
**DW** (g plant^-1^)	0.15 ± 0.01c	0.06 ± 0.00 a	0.21 ± 0.03 d	0.12 ± 0.01 b
**Rate of photosynthesis** (µmol CO_2_ m^-2^ s^-1^)	15.63 ± 0.75 c	8.65 ± 1.59a	19.46 ± 1.1 d	10.67 ± 0.86 b
**Total chlorophylls** (mg g^-1^ FW)	0.34 ± 0.03 c	0.09 ± 0.02 a	0.44 ± 0.09 d	0.18 ± 0.04 b
**Chlorophyll fluorescence** (Fv/Fm)	0.79 ± 0.08 c	0.57 ± 0.02 a	0.85 ± 0.05 d	0.65 ± 0.02 b
**Stomatal conductance** (mmol m^-2^ s^-1^)	257.04 ± 12.13 c	175.18 ± 10.51 b	219.06 ± 19.7 c	151.49 ± 6.57 a
**Rubisco activity** (nmol 3-PGA mg protein^-1^ min^-1^)	69.89 ± 1.7 c	49.09 ± 1.06 a	75.67 ± 2.78 c	55.12 ± 2.2 b

Values are presented as the mean of five independent replicates ± standard error. Means followed by similar lower-case letters in the same row are not significantly different at the 0.05 probability level as indicated by Tukey’s multiple range tests.

### 3.2 Accumulation of Nd and stress markers

**Figure 1:**
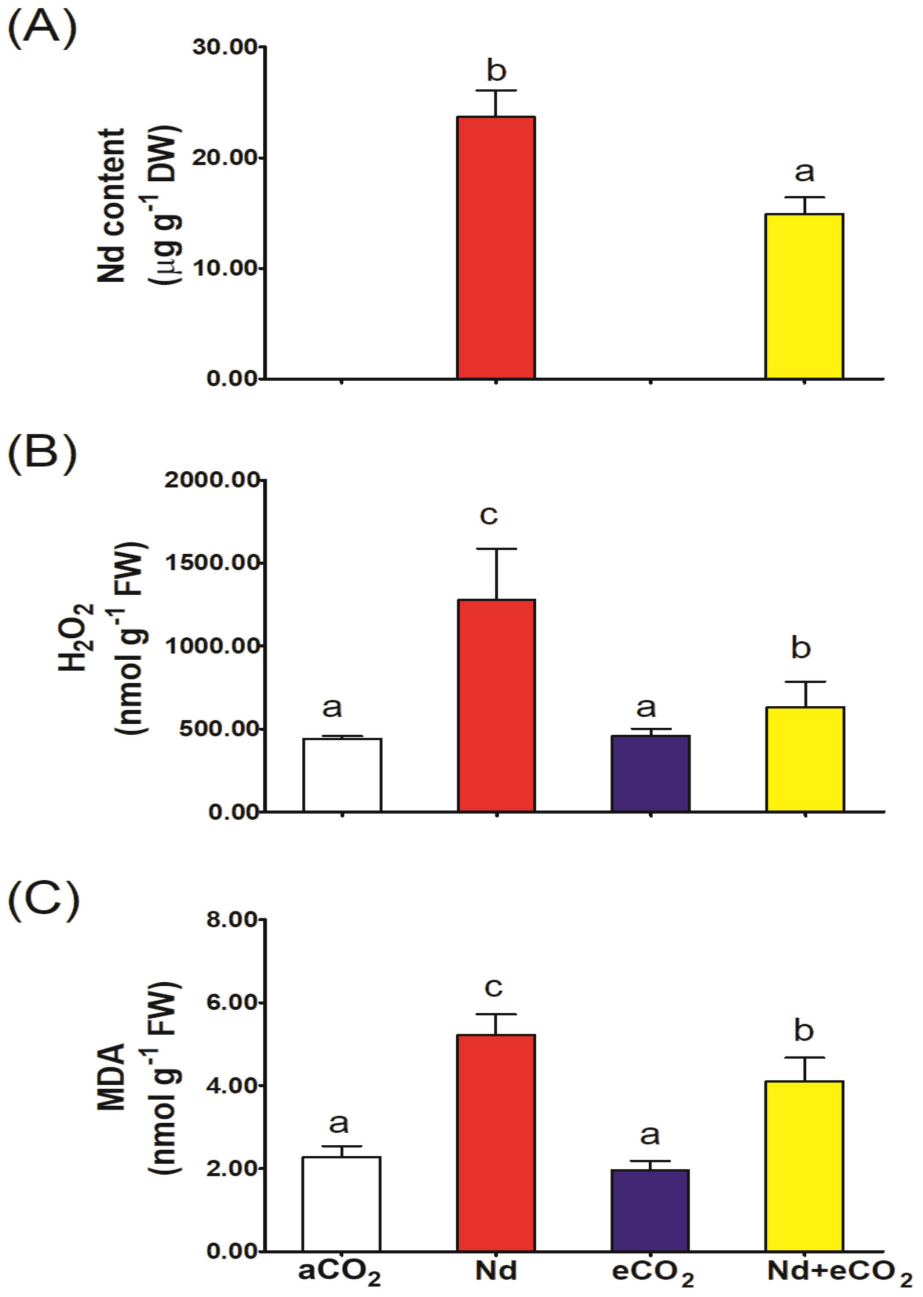
Effect of neodymium (Nd), elevated CO_2_ (eCO_2_) and their combination (Nd+eCO_2_) on the accumulation of Nd and oxidative stress markers in shoots of 4-weeks old *wheat (Triticum aestivum)* plants. **(A)** Nd concentration, mg g^-1^ DW; **(B)** H_2_O_2_, nmol g^-1^ FW; **(C)** malondialdehyde, MDA nmol g^-1^ FW. Each value is the mean of 5 independent replicates and the vertical bars demonstrates the standard error. Similar lower-case letters on the bars, within the same graph, indicate non-significant difference at the 0.05 probability level as indicated by Tukey’s multiple range tests.

As expected, application of Nd sharply increased the accumulation of Nd in plant shoots (**Figure 1A**); however, Nd accumulation was significantly reduced (~50%) at elevated [CO_2_]. Nd treatment induced about 3-fold accumulation of H_2_O_2_ and a two-fold increase in the level of MDA (**Figures 1B, C**). Elevated [CO_2_] per se had no significant impact on H_2_O_2_ and MDA levels compared to the control. Interestingly, the synchronous application of eCO_2_ with Nd significantly reduced the production of H_2_O_2_ and MDA by about 50 and 22%, respectively, compared to Nd treatment alone. A significant interaction effect was noted for H_2_O_2_ and MDA (**Supplementary Data Sheet S1**).

### 3.3 Molecular antioxidants and total antioxidant capacity

**Figure 2:**
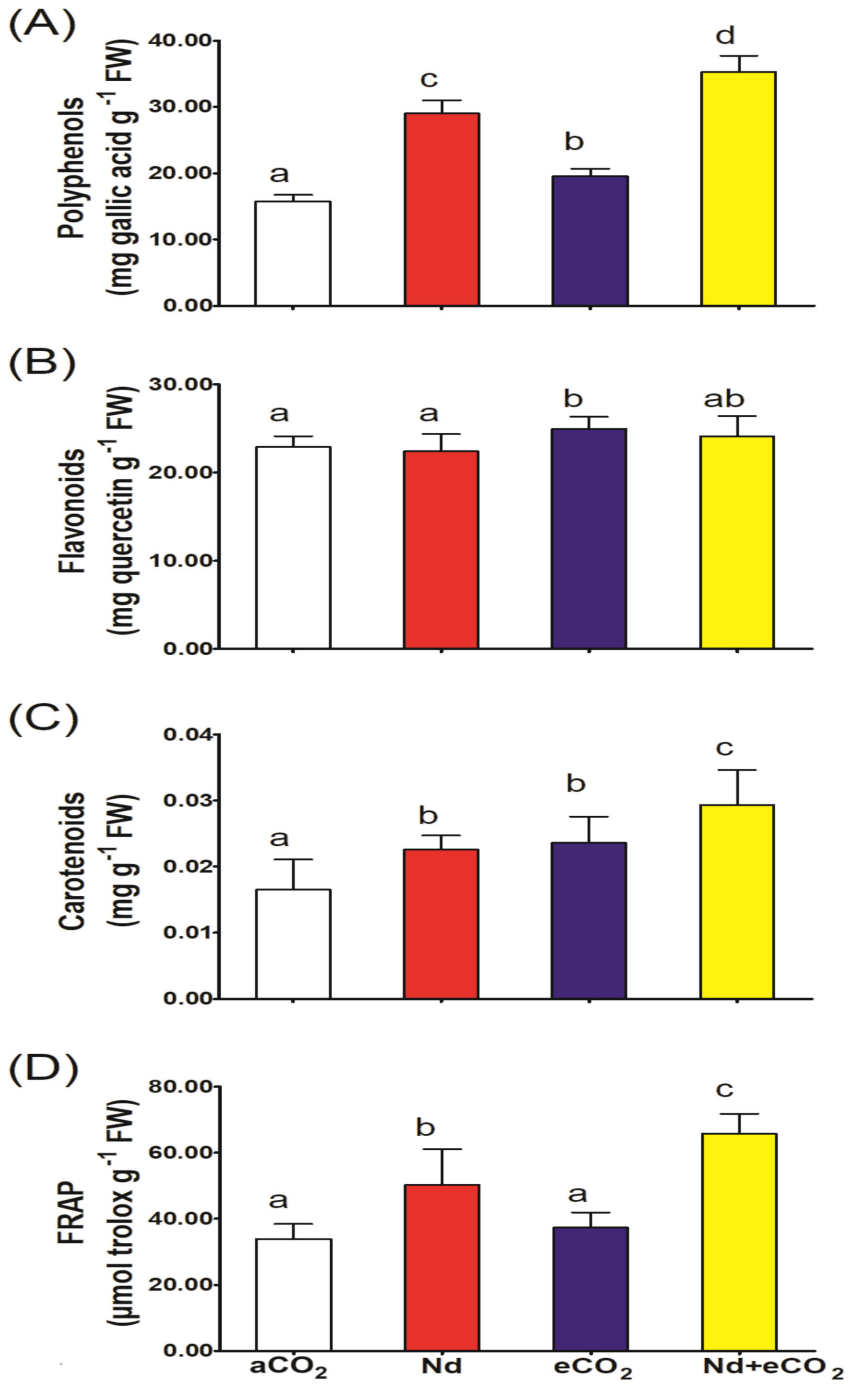
Effect of neodymium (Nd), elevated CO_2_ (eCO_2_) and their combination (Nd+eCO_2_) on the accumulation of molecular antioxidants and total antioxidant capacity (FRAP) in shoots of 4-weeks old *wheat (Triticum aestivum)* plants. **(A)** polyphenols, mg gallic acid g^-1^ FW; **(B)** flavonoids, mg quercetin g^-1^ FW; **(C)** carotenoids, mg g^-1^ FW; **(D)** FRAP mmol troloxg^-1^ FW. Each value is the mean of 5 independent replicates and the vertical bars demonstrates the standard error. Similar lower-case letters on the bars, within the same graph, indicate non-significant difference at the 0.05 probability level as indicated by Tukey’s multiple range tests.

Wheat plants treated with Nd only significantly accumulated polyphenols but not flavonoids, when compared to the control. However, plants subjected to eCO_2_ under Nd-free conditions contained significantly higher levels of flavonoids and total polyphenols (**Figures 2A, B**). Both Nd and elevate [CO_2_] significantly increased the content of carotenoids and total antioxidant capacity (FRAP) in wheat plants (**Figures 2C, D**) relative to Nd per se.

### 3.4 Antioxidants enzymes and glutathione-S-transferase

**Figure 3:**
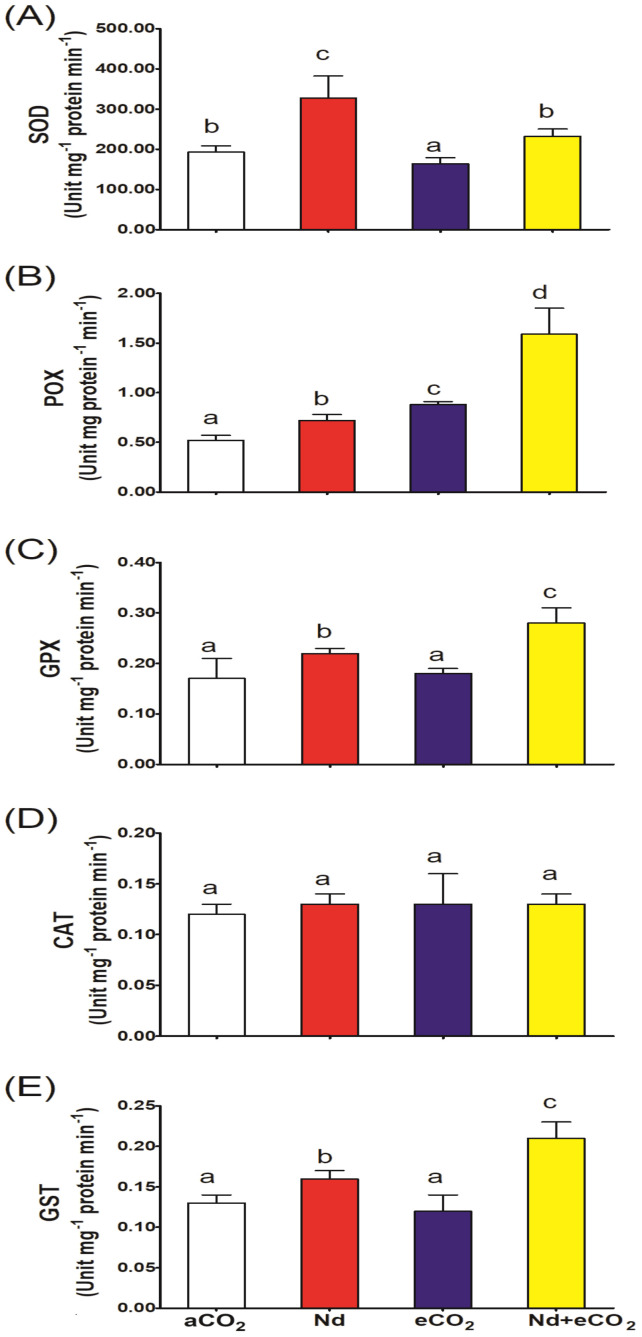
Effect of neodymium (Nd), elevated CO_2_ (eCO_2_) and their combination (Nd+eCO_2_) on the activities of antioxidant and detoxification enzymes (unit mg^-1^ protein min^-1^) in shoots of 4-weeks old *wheat (Triticum aestivum)* plants. **(A)** SOD, superoxide dismutase; **(B)** POX, peroxidase; **(C)** GPX, glutathione peroxidase; **(D)** CAT, catalase; **(E)** GST, glutathione-S-transferase. Each value is the mean of 5 independent replicates and the vertical bars demonstrates the standard error. Similar lower-case letters on the bars, within the same graph, indicate non-significant difference at the 0.05 probability level as indicated by Tukey’s multiple range tests.

Neodymium treatment upregulated the activities of SOD, POX and GPX by about 69, 38 and 29%, respectively, relative to the control (**Figures 3A–C**). On the other hand, eCO_2_ alone treatment decreased the activity of SOD and had no impact on GPX. No significant variation was detected in the catalase activity in response to either treatment (**Figure 3D**). GST activity was significantly enhanced (23%) in response to Nd treatment, but not eCO_2_ (**Figure 3E**). The activities of POX, GPX and GST showed further improvements in response to the synchronous application of eCO_2_ and Nd (120, 27 and 31%, respectively), compared to the Nd alone treatment.

### 3.5 Metabolites and enzymes of ascorbate-glutathione cycle

**Figure 4:**
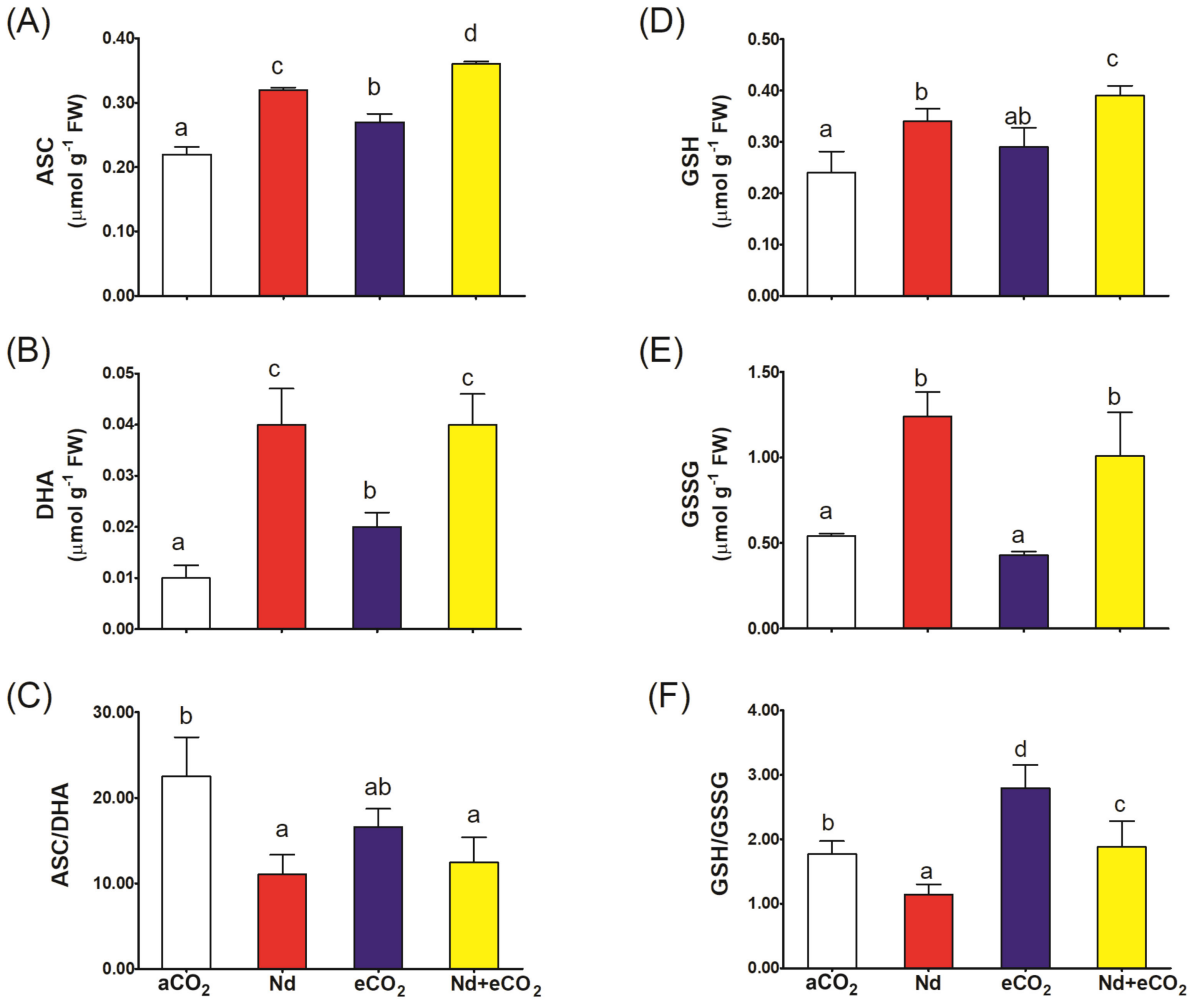
Effect of neodymium (Nd), elevated CO_2_ (eCO_2_) and their combination (Nd+eCO_2_) on the levels of the metabolites of ascorbate-glutathione (ASC-GSH) cycle (µmol g^-1^ FW) in shoots of 4-weeks old *wheat (Triticum aestivum)* plants. **(A)** ASC, reduced ascorbate; **(B)** DHA, oxidized ascorbate; **(C)** ASC/DHA ratio; **(D)** GSH, reduced glutathione; **(E)** GSSG, oxidized glutathione; **(F)** GSH/GSSG ratio. Each value is the mean of 5 independent replicates and the vertical bars demonstrates the standard error. Similar lower-case letters on the bars, within the same graph, indicate non-significant difference at the 0.05 probability level as indicated by Tukey’s multiple range tests.

**Figure 5:**
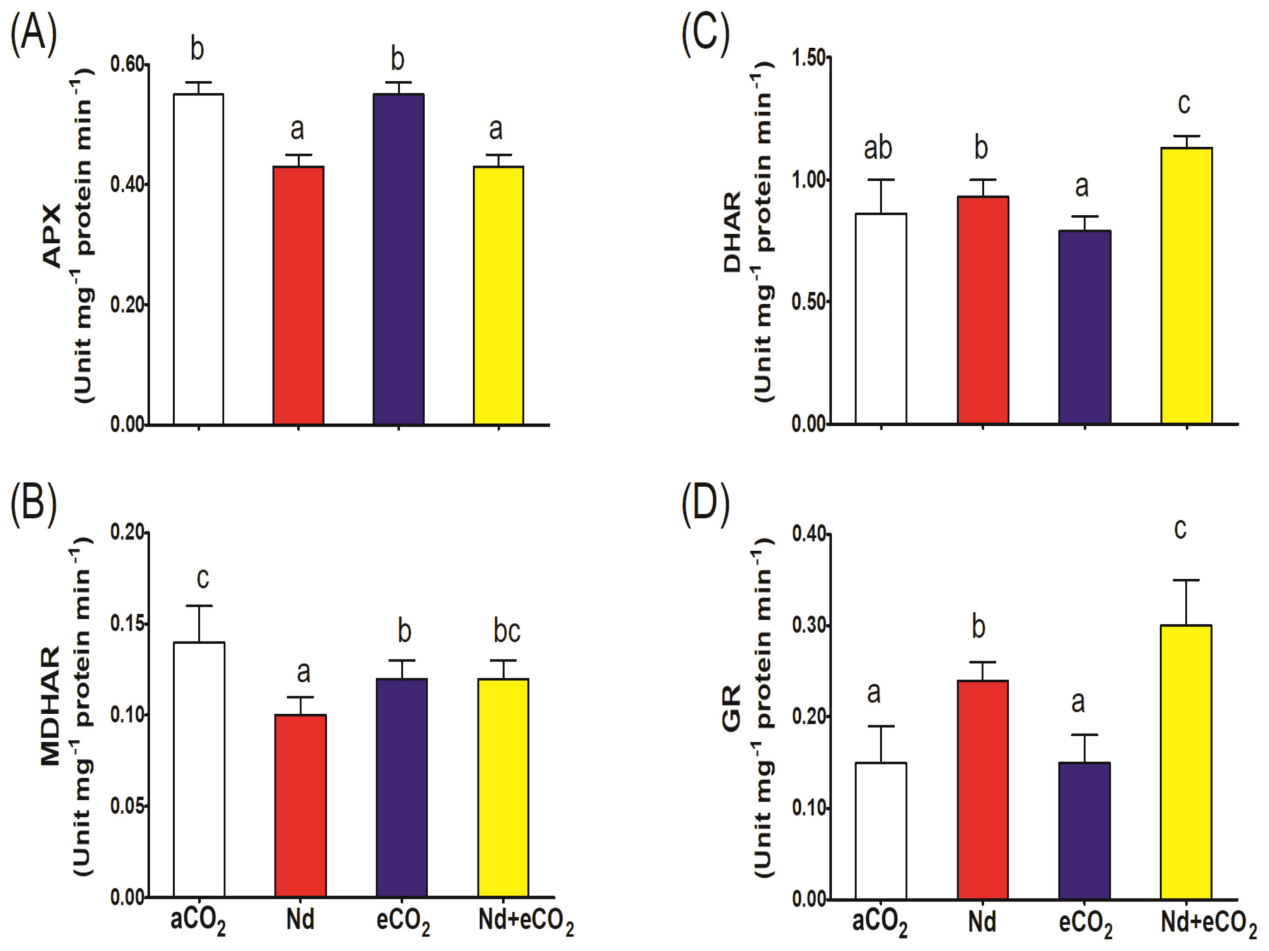
Effect of neodymium (Nd), elevated CO_2_ (eCO_2_) and their combination (Nd+eCO_2_) on the activities of the enzymes of ascorbate-glutathione (ASC-GSH) cycle (unit mg^-1^ protein min^-1^) in shoots of 4-weeks old *wheat (Triticum aestivum)* plants. **(A)** APX, ascorbate peroxidase; **(B)** MDHAR, monodehydroascorbate reductase; **(C)** DHAR, dehydroascorbate reductase; **(D)** GR, glutathione reductase. Each value is the mean of 5 independent replicates and the vertical bars demonstrates the standard error. Similar lower-case letters on the bars, within the same graph, indicate non-significant difference at the 0.05 probability level as indicated by Tukey’s multiple range tests.

Nd treatment per se increased the accumulation of all metabolites of the ASC-GSH cycle relative to the control (**Figure 4**). However, the impact of Nd was much more evident on the oxidized forms where it increased DHA and GSSG levels 4x and 2.5x, respectively (**Figures 4B, E**). Accordingly, Nd stress resulted in sharp reduction in the ASC/DHA and GSH/GSSG redox balances (**Figures 4C, F**). Such Nd-induced negative impact was associated with inhibition in the activities of ASC-GSH cycling enzymes (APX, DHR, MDHAR and GR; **Figures 5A–D**). On the other hand, eCO_2_ alone had no significant impact on the ASC/DHA ratio but improved that of GSH/GSSG (59%). If additional CO_2_ is provided, the levels of ASC and GSH increased (13 and 15%, respectively) and the activities of DHAR, MDHAR and GR increased (22, 20 and 25%, respectively) when compared to their respective values in Nd alone treated plants. In addition, eCO_2_ resulted in a significant elevation in the GSH/GSSG redox balance (65%) in Nd-stressed plants.

## 4 Discussion

### 4.1 Elevated CO_2_ can mitigate the negative impact of Nd on wheat growth and photosynthesis

The current results highlight that Nd soil contamination can notably hinder the early growth of wheat seedlings. Although the phytotoxic effects of Nd are not fully understood, they are believed to be concentration-dependent. At low to moderate concentrations, Nd has been shown to promote plant growth, including that of wheat and rice (Luo et al., 2008; Rezaee et al., 2018). However, at higher concentrations (10 and 25 mg l⁻¹ Nd₂O₃), Nd exhibits inhibitory effects on plant growth (Basu and Dhal, 2016).

Similar behavior was reported in *Helianthus annuus* and *Brassica chinensis* subjected to different levels of Nd (Rezaee et al., 2018; Rezaee, 2016). The phytotoxic effects of Nd were reported at various concentrations depending on plant species and environmental conditions. Currently 200 mg Nd^3+^kg^-1^ soil indicated adverse effect on wheat where Carpenter et al. (2015) reported that Nd at 319 mg/kg were significantly toxic and adversely affected plant growth and overall biomass when studied some native and crop species. Zhao et al. (2019) also assessed the effects of different concentrations of Nd on the growth of various crop species. It was found that concentrations as low as 100 mg/kg of Nd resulted in notable reductions in root development of wheat.

The reduction in plant fresh and dry biomass is a logical consequence of the phytotoxicity of metals on the important physiological processes, e.g., photosynthesis and nutrient utilization (Malik et al., 2019). Like most REEs, Nd may interfere with the key plant metabolic processes and components such as rate of photosynthesis, total chlorophylls, chlorophyll fluorescence, stomatal conductance and rubisco activity since all were significantly decreased in relation to control. In this regard, Rezaee (2016) concluded that the inhibition in chlorophyll biosynthesis in *B. chinensis* started at Nd concentration of 200 ppm. Nd exposure has been shown to disrupt chlorophyll fluorescence in *Myriophyllum aquaticum* coupled with significant chlorophyll degradation (Gjata et al., 2024). Furthermore, exposure to Nd also inhibited rubisco enzyme activity, a critical component in carbon fixation during photosynthesis which is in accordance with report about Cd and Cu toxicity in tobacco plants (Khairy et al., 2016). As one of REEs, lanthanum disrupts photosynthesis and affects the over whole plant health (Jiao et al., 2024). Exposure to Nd has been demonstrated to disrupt chlorophyll fluorescence in *Myriophyllum aquaticum*, leading to significant chlorophyll degradation (Gjata et al., 2024). Additionally, Nd exposure inhibits the activity of the rubisco enzyme, which is essential for carbon fixation during photosynthesis, similar to the effects of cadmium (Cd) and copper (Cu) toxicity observed in tobacco plants (Khairy et al., 2016). As a member of the rare earth elements (REEs), lanthanum also interferes with photosynthesis, impacting overall plant health (Jiao et al., 2024). Currently elevated [CO_2_] by itself significantly improved the growth and photosynthesis in wheat, which is consistent with the report of Kang et al. (2021) when investigated wheat and rice. Further, the aerial growth and photosynthetic rates were increased as consequences to eCO_2_ in oak seedlings (Sanchez-Luca et al., 2023). Synchronous application of eCO_2_ and HMs was reported to lessen the toxic effects of HMs on plant growth and yield, especially in C3 plant systems, through inducing the synthesis of chlorophyll and its fluorescence, stomatal conductance, and net photosynthetic rate (Habeeb et al., 2020; Saleh et al., 2021). It is of interest to note here that additional CO_2_ only partially relieved the negative impact of Nd on plant crucial processes. Similarly, eCO_2_ improved the biomass production and photosynthesis of wheat and antagonized the negative impact of NiO nanoparticles and Al on these processes (Saleh et al., 2019; AbdElgawad et al., 2021). To date, there are no previous studies on the interactive effects of Nd and eCO_2_ on plants. However, the ameliorative effect of eCO_2_ on HM toxicity is not universal and depends on the specific HM and plant species. Current data therefore highlights a significant interaction between Nd and Elevated [CO_2_], where the beneficial effects of Elevated [CO_2_] are partially offset by the presence of Nd, suggesting the need for further research into the mechanisms of this interaction.Top of FormBottom of Form

### 4.2 Accumulation of Nd in wheat plants induces less oxidative damage under eCO_2_ environment

The combined effect of Nd and elevated [CO_2_] can help mitigate the oxidative stress typically induced by Nd accumulation in wheat plants. This interaction is crucial for understanding how these conditions affect plant health and productivity. Wheat plants grown in NdCl_3_ contaminated soil accumulated high levels of Nd in their shoots and showed the highest levels of H_2_O_2_ and MDA. Similar results were reported in lentil seedlings treated with Nd (Gjata et al., 2022). Hyperaccumulation of H_2_O_2_ and MDA in plants is an evident sign for severe oxidative cellular damage. Indeed, it has been reported that REEs could alter the redox activity and plasma membrane permeability of plant cells, which can lead to membrane disfunction and increased ROS and MDA levels (Oral et al., 2017; Peng et al., 2007; Zicari et al., 2018). Consistent with the current results, elevated levels of H_2_O_2_ and MDA were recorded in onion plants treated with Nd (Gjata et al., 2022). It suggests that the reactive oxygen species (ROS) production is a common plant response suffer metal stress conditions.

Interestingly, elevated [CO_2_] reduced the accumulation of Nd in wheat plants and, therefore, the levels of oxidative stress markers, H_2_O_2_ and MDA were also reduced. Lower Nd levels could be associated with eCO_2_ induced reductions in stomatal conductance and lower Nd uptake through transportational flow. In addition, elevated [CO_2_] is known to downregulate photorespiration, a major H_2_O_2_-producing process in plants that is enhanced during oxidative stress (Foyer and Noctor, 2000). The decline in Nd observed here is consistent with other studies in wheat showing that elevated [CO_2_] can reduce the uptake and accumulation of Ni (Saleh et al., 2019) and Al (AbdElgawad et al., 2021). In addition, the effect of eCO_2_ on photorespiration and ROS production in wheat and other crops has been previously reported (Saleh et al., 2019, 2021; AbdElgawad et al., 2022b). Indium oxide nanoparticles induced the H_2_O_2_, lipid peroxidation levels which has been mitigated by elevated [CO_2_] in maize and barely (Shabbaj et al., 2022).

To compensate for Nd-induced ROS production, wheat plants synthesized more polyphenols, and carotenoids, which are integrated into TAC, i.e., FRAR. Similarly, wheat plants suffering HMs-induced oxidative stress were reported to accumulate various molecular antioxidants (Saleh et al., 2019, 2021). Application of elevated [CO_2_], either alone or in conjunction with Nd, also induced more polyphenols and carotenoids. The upregulation of molecular antioxidants in plants grown in elevated [CO_2_] environments, under either HMs free or stressed conditions, have been previously reported (AbdElgawad et al., 2023; Al Jaouni et al., 2018; Saleh et al., 2018).

Overproduction of ROS can be compensated, in part, by stimulating the production of antioxidizing and detoxifying enzymes. This was observed in the current study with noted increases in SOD, POX, GPX and GST in Nd-treated plants. This stimulation has also been observed for SOD and POD enzymes in spinach, wheat and rice plants treated with Nd (Chao et al., 2009; Shi et al., 2021).

Elevated [CO_2_] also stimulated the activities of POX, GPX and GST which could explain the decreased levels of H_2_O_2_ and MDA in Nd + eCO_2_ treated plants, compared to Nd alone. Similarly, Elevated [CO_2_] was reported to upregulate the enzymatic defense pool in several plant species, including wheat, under HMs stressed conditions (Saleh et al., 2019, 2021; AbdElgawad et al., 2021, 2022b, 2022a).

Besides the higher concentrations of H_2_O_2_ and MDA, declines in GSH/GSSG and ASC/DHA redox balances were recorded in Nd-treated plants. Such reductions in GSH/GSSG and ASC/DHA redox balances could be explained by the negative impact of Nd on ASC-GSH recycling enzymes, e.g., APX and MDHAR. Similarly, 25 μM Nd was reported to decrease the ASC/total ASC ratio in roots of lentil seedlings (Gjata et al., 2022). Elevation of GSH/GSSG ratio in response to eCO_2_, as a result for upregulating the activities of DHAR and GR, indicates its ability to adapt to the oxidative damage induced by Nd. Several studies have reported the positive impact of eCO_2_ on ASC-GSH recycling in plants subjected to HMs (Saleh et al., 2019, 2021; AbdElgawad et al., 2021, 2022b, 2022a). The interaction between Nd and eCO_2_ showed that elevated CO_2_ have high effects on plant response under Nd stress. Significant interactions of Nd and eCO_2_ were observed for most of the investigated parameters. Such findings suggested that the combined Nd and elevated CO_2_ may trigger antioxidant defences system that may help to ameliorate some of the negative impacts of Nd by enhancing the plant’s antioxidant capacity. Further research into the ecological implications of REE accumulation in agricultural systems, especially under changing atmospheric conditions such as increased CO_2_ is needed.

## 5 Conclusion

The current study provides the first comprehensive analysis of oxidative stress in wheat caused by neodymium (Nd) and explores the potential adaptive benefits of elevated atmospheric CO_2_ levels. While increased CO_2_ concentrations do not completely mitigate the growth reductions induced by Nd, they do alleviate some oxidative stress. Potential strategies for mitigating the effects of Nd under elevated CO_2_ include: 1) reducing Nd uptake and accumulation in photosynthetic tissues while lowering stomatal conductance; 2) mitigating the negative impact of Nd on photosynthetic carbon assimilation by enhancing photochemical efficiency and Rubisco activity; and 3) minimizing the increase in reactive oxygen species (ROS) generated by Nd at both production and elimination stages. Although these findings are promising, they remain preliminary, and further research is essential to fully understand the metabolic interactions between elevated CO_2_ and rare earth elements (REEs).

## Data Availability

The original contributions presented in the study are included in the article/**Supplementary Material**. Further inquiries can be directed to the corresponding author.
