# Supplementary material for: Carbon dioxide-enriched atmosphere diminished the phytotoxicity of neodymium in wheat (Triticum aestivum L.)
Source: Front Plant Sci. 2025 Apr 28;16:1521460. doi: 10.3389/fpls.2025.1521460 (PMC12076478; doi:10.3389/fpls.2025.1521460)
Supplement: Supplementary file 1 [file DataSheet1.docx]

**Carbon dioxide-enriched atmosphere diminished the phytotoxicity of neodymium in wheat (*Triticum aestivum* L.)**

**Supplementary data, S1**: A two-way ANOVA for the effect of neodymium (Nd), elevated CO2 (eCO2) interaction on the assessed parameters in shoots of 4-weeks old wheat (*Triticum aestivum*) plants. Numbers represent F values; ns = non-significant; *=*P* < 0.05; **=*P* < 0.01; *** = *P* < 0.001.

| **Parameter** | **Nd** | | **eCO2** | | **Nd X eCO2** | |
| --- | --- | --- | --- | --- | --- | --- |
|  | **F** | **Sig.** | **F** | **Sig.** | **F** | **Sig.** |
| FW | 169.190 | *** | 29.754 | *** | 5.923 | * |
| DW | 120.333 | *** | 40.333 | *** | 8.790 | ** |
| Photosynthesis | 56.533 | *** | 11.162 | * | 10.194 | * |
| CHLa | 58.156 | *** | 6.395 | * | 4.014 | ns |
| CHLb | 181.504 | *** | 8.539 | ** | 6.258 | * |
| CHLab | 83.913 | *** | 6.311 | * | 5.429 | * |
| Carotenoids | 5.247 | * | 8.069 | * | 34.993 | *** |
| gs | 5.392 | * | 1.101 | ns | 10.032 | ** |
| CHLflourec | 26.695 | *** | 2.609 | ns | 3.109 | ns |
| Rubisco | 143.482 | *** | 11.089 | ** | .076 | ns |
| POX | 31.869 | *** | 63.687 | *** | 12.367 | ** |
| CAT | 1.006 | ns | .017 | ns | 4.171 | ns |
| SOD | 15.216 | ** | 8.565 | ** | 37.029 | *** |
| APX | 30.534 | *** | .000 | ns | 0.000 | ns |
| DHAR | 15.235 | ** | 2.105 | ns | 14.906 | ** |
| MDHAR | 5.327 | * | 1.066 | ns | 4.148 | ns |
| GR | 12.877 | ** | 1.379 | ns | 1.939 | ns |
| GPX | 11.167 | * | 3.824 | ns | 5.253 | * |
| GST | 12.457 | ** | 2.690 | ns | 10.944 | ** |
| HMcontent | 323.927 | *** | 13.994 | .006 | 13.994 | ** |
| H2O2 | 26.763 | *** | 11.014 | .011 | 11.934 | ** |
| MDA | 31.629 | *** | 1.451 | ns | 48.046 | *** |
| FRAP | 13.324 | ** | .794 | ns | 6.267 | * |
| Polyphenol | 129.823 | *** | 15.302 | .004 | 0.914 | ns |
| Flavo | 1.093 | ns | 2.345 | ns | 0.021 | ns |
| ASC | 30.933 | *** | 8.077 | .022 | 0.156 | ns |
| TASC | 24.412 | *** | 5.008 | ns | 0.159 | ns |
| DHA | 4.850 | ns | .171 | ns | 9.152 | ** |
| ASC_DHA | 1.887 | ns | .247 | ns | 36.236 | *** |
| GSH | 18.854 | ** | 6.210 | .037 | 9.543 | ** |
| TGSH | 32.315 | *** | .282 | ns | 81.929 | *** |
| GSSG | 15.413 | ** | 1.295 | ns | 131.102 | *** |
| GSH_GSSG | 3.202 | ns | 4.726 | ns | 42.503 | *** |
